# Functional analysis of the zebrafish ortholog of *HMGCS1* reveals independent functions for cholesterol and isoprenoids in craniofacial development

**DOI:** 10.1371/journal.pone.0180856

**Published:** 2017-07-07

**Authors:** Anita M. Quintana, Jose A. Hernandez, Cesar G. Gonzalez

**Affiliations:** 1 Department of Biological Sciences, University of Texas El Paso, El Paso, TX, United States of America; 2 Border Biomedical Research Center, NeuroModulation Cluster, University of Texas El Paso, El Paso, TX, United States of America; Texas A&M University, UNITED STATES

## Abstract

There are 8 different human syndromes caused by mutations in the cholesterol synthesis pathway. A subset of these disorders such as Smith-Lemli-Opitz disorder, are associated with facial dysmorphia. However, the molecular and cellular mechanisms underlying such facial deficits are not fully understood, primarily because of the diverse functions associated with the cholesterol synthesis pathway. Recent evidence has demonstrated that mutation of the zebrafish ortholog of *HMGCR* results in orofacial clefts. Here we sought to expand upon these data, by deciphering the cholesterol dependent functions of the cholesterol synthesis pathway from the cholesterol independent functions. Moreover, we utilized loss of function analysis and pharmacological inhibition to determine the extent of sonic hedgehog (Shh) signaling in animals with aberrant cholesterol and/or isoprenoid synthesis. Our analysis confirmed that mutation of *hmgcs1*, which encodes the first enzyme in the cholesterol synthesis pathway, results in craniofacial abnormalities via defects in cranial neural crest cell differentiation. Furthermore targeted pharmacological inhibition of the cholesterol synthesis pathway revealed a novel function for isoprenoid synthesis during vertebrate craniofacial development. Mutation of *hmgcs1* had no effect on Shh signaling at 2 and 3 days post fertilization (dpf), but did result in a decrease in the expression of *gli1*, a known Shh target gene, at 4 dpf, after morphological deficits in craniofacial development and chondrocyte differentiation were observed in *hmgcs1* mutants. These data raise the possibility that deficiencies in cholesterol modulate chondrocyte differentiation by a combination of Shh independent and Shh dependent mechanisms. Moreover, our results describe a novel function for isoprenoids in facial development and collectively suggest that cholesterol regulates craniofacial development through versatile mechanisms.

## Introduction

Craniofacial malformations are a group of heterogeneous disorders that describe developmental defects of the head and facial bones. The most common forms of craniofacial abnormalities include cleft lip/palate, craniosynostosis, and facial dysostoses. The prevalence of such disorders encompasses over 1/3 of all congenital malformations [1]. The elements of the face are derived primarily from neural crest cells (NCC), a pluripotent stem cell population formed at the dorsal end of the neural tube upon neural tube closure [2]. While many factors have been shown to modulate NCC function and craniofacial development, the presence of dysmorphic features in Smith-Lemli-Opitz syndrome (SLOS) and other known disorders of cholesterol [3,4] metabolism strongly suggests that the cholesterol synthesis pathway regulates NCC development.

The cholesterol synthesis pathway produces two separate, but equally important molecules: cholesterol, a common membrane component, and isoprenoids, molecules important for protein prenylation and cell signaling. The first step of the pathway is mediated by *HMGCS1*, the gene that encodes HMG-CoA synthase, which converts acetyl CoA and acetoacetyl-CoA into 3-hydroxy-3-methylglutaryl CoA (HMG-CoA). HMG-CoA is then reduced by HMG-CoA reductase (encoded by *HMGCR*) into mevalonate in the rate limiting step of the reaction. The subsequent production of farnesyl pyrophosphate represents a branch point in the pathway, serving as a substrate for either cholesterol or isoprenoid synthesis.

Recently the presence of craniofacial abnormalities was documented in a zebrafish mutant of *hmgcrb*, the ortholog of *HMGCR* [5]. Zebrafish are uniquely powerful in this context as equivalent mutations in mice are embryonically lethal [6]. Mutation of *hmgcrb* in zebrafish resulted in a shortening of the ethmoid plate and/or the complete loss of specific cartilage elements. However, mutation or inhibition of *HMGCR* inhibits the synthesis of not only cholesterol, but also isoprenoids. Thus, despite significant functional analysis in *hmgcrb* mutations, we still do not understand if the mechanisms underlying the facial phenotype in *hmgcrb* mutants are cholesterol dependent, cholesterol independent, or a combination of both.

Isoprenoids represent a large class of sterol molecules whose functions span the electron transport chain, hormone synthesis, and protein prenylation/signal transduction [7]. Furthermore, inhibition of the cholesterol synthesis with statins, which interfere with the activity of HMGCR, cause pleiotropic effects, many of which are thought to be a consequence of defective isoprenoid synthesis [8]. Despite the heterogeneous functions associated with isoprenoids, the relationship between their synthesis and craniofacial development has been largely unexplored. Importantly, recent evidence utilizing a zebrafish mutant of *hmgcs1* in combination with pharmacological inhibition was used to demonstrate discrete functions for isoprenoids and cholesterol during central nervous system myelination [9]. Together these data provide strong evidence that zebrafish are a powerful model to understand the independent functions of isoprenoids and cholesterol.

Many of the mechanisms underlying facial dysmorphia in SLOS and related disorders have been attributed to either defects in cholesterol or the accumulation of sterols [10]. It has been proposed that those mechanisms related to cholesterol deficiency are primarily mediated through the sonic hedgehog (Shh) signaling pathway because disorders of cholesterol metabolism share phenotypic features with genetic disorders of Shh signaling [11–13]. Moreover, the post-translational modification of Shh by cholesterol is essential for appropriate Shh activation [14,15]. However, a direct link between deficiencies in cholesterol, Shh inactivation, and facial defects has not been completely elucidated *in vivo*.

In this study we sought to determine the independent functions of cholesterol and isoprenoids by combining genetic loss of function assays with pharmacological inhibition. We found that mutation of *hmgcs1* results in craniofacial abnormalities, including a distinct truncation of the Meckel’s cartilage and the absence of ceratobranchial cartilages, a finding that is consistent with previously published reports [5]. Since mutations in *hmgcs1* inhibit the synthesis of isoprenoids as well as cholesterol, we further sought to understand the unique contributions of each lipid molecule using pharmacological inhibition. Our data confirm that deficiencies in cholesterol biosynthesis interfere with vertebrate craniofacial development, but in addition, we demonstrate a novel function for isoprenoids in NCC differentiation and facial development. Moreover, we analyzed the activity of Shh in *hmgcs1* mutants and embryos with pharmacologically induced deficiencies in cholesterol or isoprenoids. Shh activity was normal for the first 3 days of development in *hmgcs1* mutants and/or drug treatment groups, yet morphological defects in craniofacial development were evident by 3 days post fertilization (dpf). These data provide strong evidence that the facial deficits present in *hmgcs1* mutant larvae are not entirely Shh dependent. Taken together our data suggest that both cholesterol and isoprenoids play complex roles in the regulation of craniofacial development.

## Material and methods

### Zebrafish maintenance and genotyping

For all experiments, embryos were obtained by crossing adult Tupfel long fin, AB, *hmgcs1*^Vu57^ [9], *Tg*(*sox10*:memRFP) [16], or *Tg*(*sox10*:tagRFP) [17,18]. Embryos were maintained in embryo medium at 28.5°C. This study was approved by the Institutional Animal Care and Use Committees at the University of Colorado Anschutz Medical Campus and The University of Texas El Paso according to the Institutional Animal Care and Use Committee (IACUC) guidelines (protocols #B-85411(08)1D or 811689–5). Genotyping of *hmgcs1*^Vu57^ was performed according to PCR amplification and restriction digest. Briefly, single nucleotide variant specific primers were designed using the dCAPS Finder 2.0 program (http://helix.wustl.edu/dcaps/dcaps.html) as previously described [9,19]. For all assays, the total number of embryos obtained from a heterozygous cross of individuals carrying the *hmgcs1*^Vu57^ allele were subjected to each individual assay and then imaged as described below. Imaged individual embryos were lysed in DNA extraction buffer (10mM Tris pH 8.2, 10mM EDTA, 200mM NaCl, 0.5% SDS, and 200ug/ml proteinase K) for 3 hours at 55°C. DNA was isolated using phenol: chloroform and ethanol precipitation. A nested PCR approach was utilized to amplify appropriate products (Outer Primers: ttacgctctggttgttgctg and gccatccaccactggatact; Inner Primers including dCAPS designed restriction site (HindIII) gatgctctgtatttatgaagatgctt and atgtccggcttataaaaatcataagc). Each PCR product was then digested with HindIII according to manufacturer’s protocol overnight at 37°C. This approach was used to identify homozygous and heterozygous siblings at all time points examined, before and after morphological phenotypes were observed. For PCR quantification of mRNA, individual embryos were anesthetized and the tail was clipped from each embryo. DNA was isolated from the tail of each individual embryo for genotyping and the head of each embryo was lysed in Trizol for RNA isolation and cDNA synthesis.

### Drug treatment

For drug treatments, Atorvastatin (Sigma, pharmaceutical grade, St. Louis MO), Lonafarnib (Sigma, St. Louis MO), and Ro 48 8071 (Santa Cruz Biotechnology, Santa Cruz, CA) were each dissolved in 100% DMSO. Drugs were diluted in embryo medium to make working solutions at the following concentrations: 2.5 uM atorvastatin, 8 uM lonafarnib, 10uM GGTI 2133, and a dose response curve from 1.5-4uM for Ro 48 8071. Final concentration of DMSO was less than 0.01% in all samples and vehicle control treatment. Drugs were added to embryos at 5 hours post fertilization (hpf) and media was changed every 18–24 hours until experimental procedure was performed. For temporal regulation studies, each drug was added at 24 hpf and removed after 24 hours of treatment.

### Cartilage staining, *in situ* hybridization, quantitative real time PCR (QPCR), and imaging

For cartilage staining, larvae were collected at 4 or 5 dpf and fixed for 1 hour in 2% paraformaldehyde (VWR, Radnor, PA) at room temperature (RT). Alcian blue was utilized to detect cartilage and performed as previously described [20] except we did not perform additional bone staining with alizarin red. The length of the Meckel’s cartilage was measured in a subset of stained fish. Quantified images were captured at equivalent magnification with a Zeiss stereo microscope and measurements were taken using the distance tool from Zen software. The distance between the top of the Meckel’s cartilage to the top of the ceratohyal was measured across all samples. Statistical significance was analyzed using a T-test. *In situ* hybridization was performed with probes and methods previously described [20–23]. Images were collected with appropriate filters using a Zeiss Discovery Stereo Microscope with Zen software. For QPCR, RNA was isolated from genotyped embryos at 2, 3, or 4 dpf. Samples for each condition were collected in duplicate. Reverse transcription was performed using iScript (Bio-Rad, Redmond, WA). QPCR was performed in triplicate for each sample using an Applied Biosystems StepOne Plus machine and associated software. Taqman assays were used to detect *patched2* (Dr03118687_m1) and *rpl13a* (Dr03101115_g1) and Sybr green was used to detect *gli1* (primers: 5’ggaggcgcatataacgaaaa3’ and 5’tcggctgagatttcagtgtg3’). Statistical analysis of mRNA expression was performed using a T-test. Fluorescent images of transgenic animals were captured using Zeiss Axiovert 200 microscope equipped with a Perkin Elmer spinning disk confocal system with Volocity software.

## Results

### Mutation of *hmgcs1* causes craniofacial abnormalities

**Fig 1:**
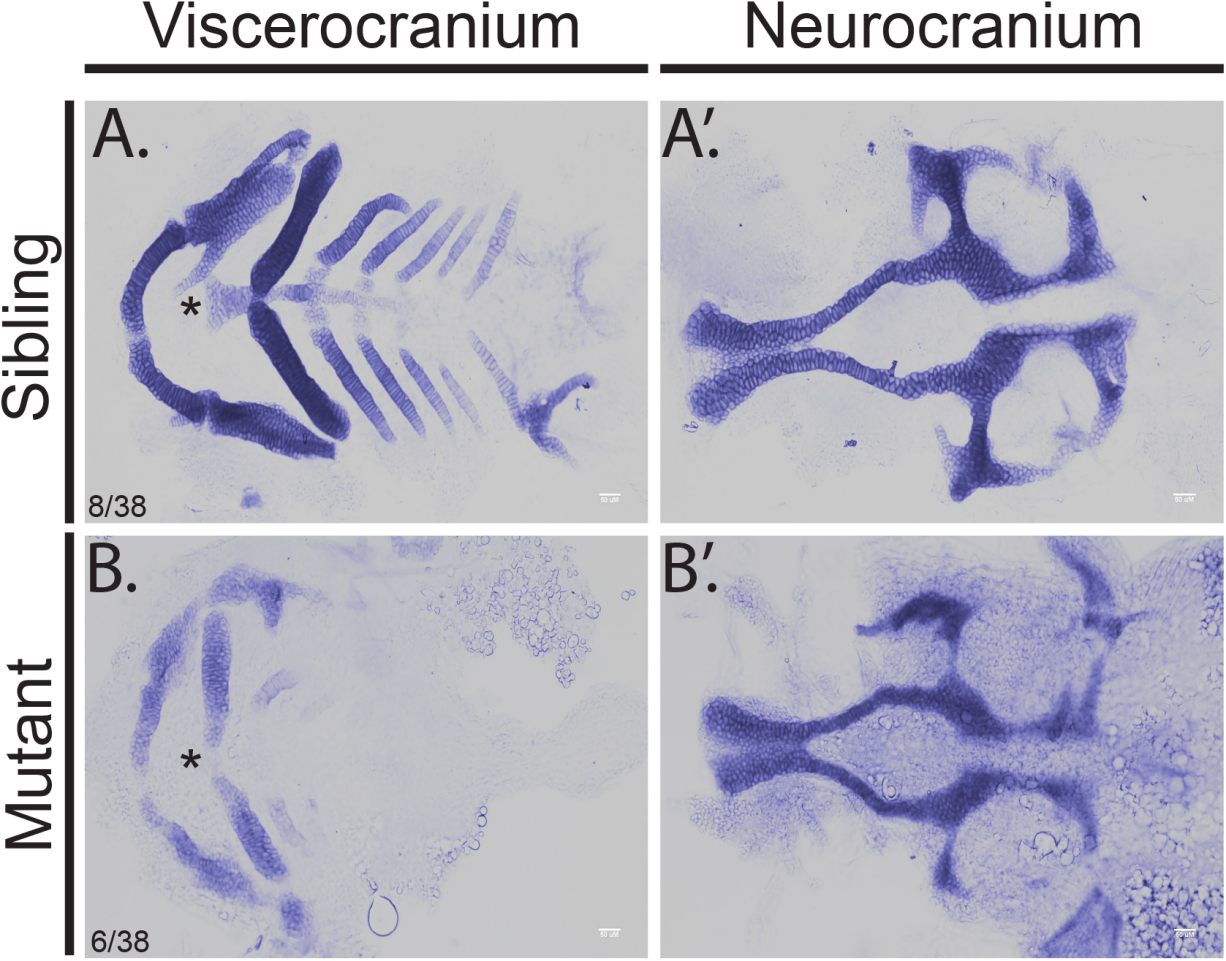
Mutation of *hmgcs1* causes craniofacial abnormalities. Alcian staining was performed to visualize the developing cartilage in *hmgcs1* wildtype or heterozygous (Sibling) or homozygous *hmgcs1* mutant larvae (Mutant). Embryos were stained at 4 days post fertilization and manual dissection of the viscerocranium and neurocranium was performed. Viscerocranium is depicted in (A) and (B) and neurocranium is depicted in (A’) and (B’). Asterisk denotes basihyal. n = 38 including 8 wildtype embryos, 6 homozygous mutants, and 24 heterozygous mutants. No significant defects were present in heterozygous carriers (S1 Fig).

Mutation of the zebrafish *hmgcrb* gene is associated with orofacial clefts [5] and therefore, we hypothesized that mutations in *hmgcs1* result in craniofacial defects. To test this hypothesis, we analyzed the facial phenotype in zebrafish harboring the *hmgcs1*^Vu57^ allele [9]. The Vu57 allele results in a p.H189Q single missense mutation in the homodimerization domain of Hmgcs1 [9]. The Vu57 allele replaces a single amino acid and is not a genetic null mutation. In order to visualize the developing cartilage structures of the face, we stained 4 dpf embryos with Alcian blue, which stains developing cartilage structures. Dissection of the viscerocranium confirmed that *hmgcs1* mutants had a truncated Meckel’s cartilage, an abnormal ceratohyal, loss of the basihyal, and malformation of ceratobranchial cartilages {Fig 1A & 1B, 8/38 wildtype siblings (WT), 6/38 homozygous mutant (MT), 24/38 heterozygous mutants (HT)}. In contrast, wildtype and heterozygous siblings had a normal Meckel’s cartilage, ceratohyal, basihyal, and the appropriate number of ceratobranchial cartilages (Figs 1A and S1). Despite these significant deficits in the developing viscerocranium, the neurocranium appeared relatively normal in *hmgcs1* mutants, including an intact ethmoid plate with appropriate formation of the trabeculae and parachordal (Fig 1A’ & 1B’). Collectively, our results are consistent with the orofacial clefts observed in *hmgcrb* mutants [5].

### Neural crest cells are produced and migrate normally in the absence *hmgcs1*

**Fig 2:**
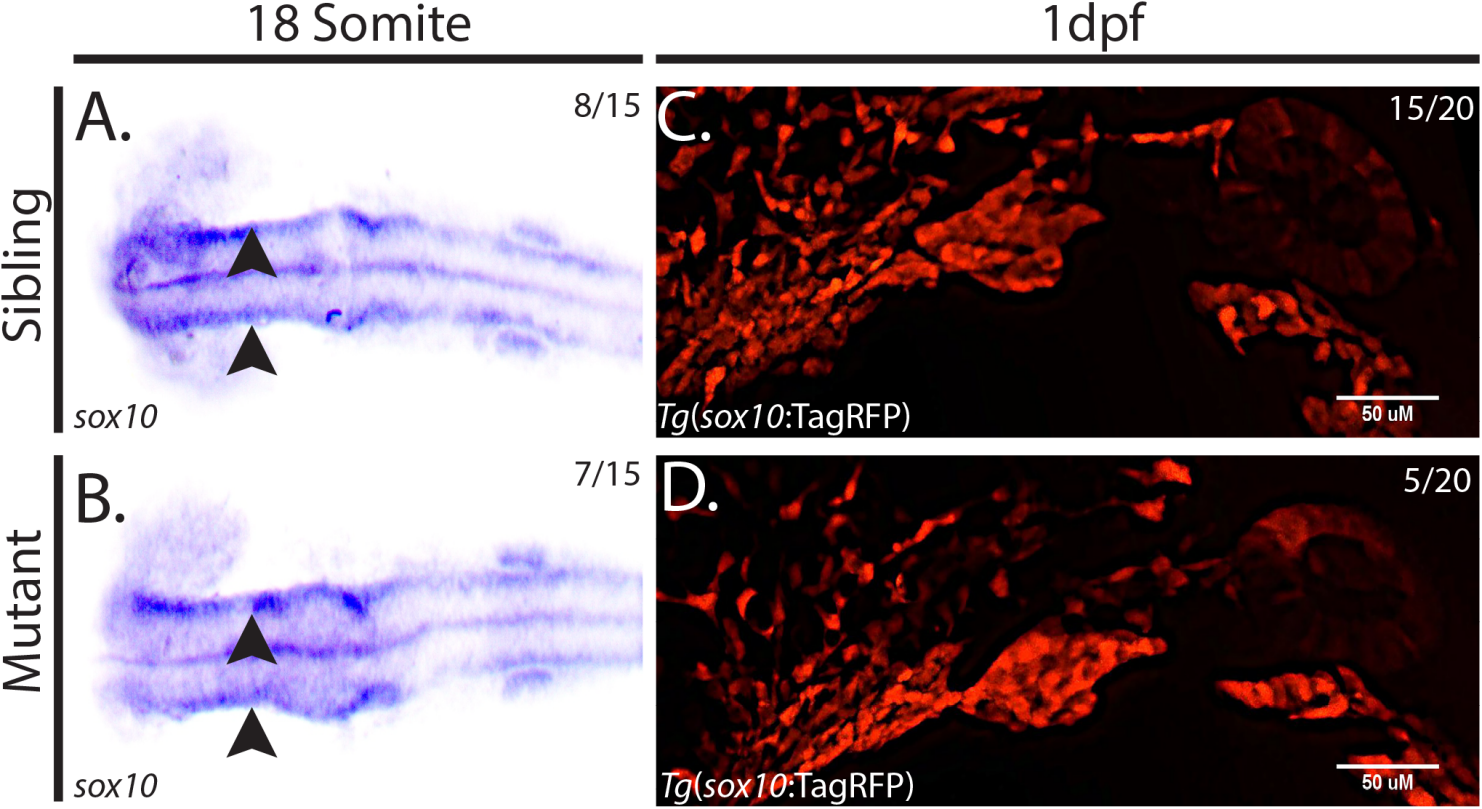
*hmgcs1* does not regulate neural crest cell specification or migration. (A&B) *In situ* hybridization to detect *sox10* expression was performed in *hmgcs1* homozygous mutants or siblings at the 18 somite stage. Dorsal view with anterior to the left. (n = 15 including 8 wildtype/heterozygous siblings and 7 homozygous mutants). Wildtype Sibling is depicted in A, but no significant differences were observed between wildtype and heterozygous carriers. Arrowheads indicated two positive streams of Sox10+ positive cells. (C&D) Sagittal view of wildtype *hmgcs1* siblings (*hmgcs1* Sibling) or *hmgcs1* homozygous mutant larvae (*hmgcs1* MT). Larvae were maintained in a *Tg*(*sox10*:tagRFP) background and RFP was visualized using traditional microscopy at 1 day post fertilization (dpf) between Prim-5 and Prim-15. n = 20 including 15 wildtype and heterozygous individuals and 5 homozygous mutant individuals. Sibling depicted is a wildtype individual. No significant difference between wildtype embryos was detected.

Defects in the number or migration of NCCs are possible mechanisms by which craniofacial deficits may arise. Therefore, we hypothesized that mutation of *hmgcs1* results in defects in NCC specification and/or migration. To test this hypothesis, we analyzed the expression of *sox10*, a transcription factor expressed in differentiating cranial NCCs [24], in animals harboring the Vu57 *hmgcs1* allele and their wildtype siblings at the 18 somite stage. *In situ* hybridization revealed two streams of *sox10* positive cells along the dorsal side of mutants and their wildtype siblings (Fig 2, n = 15 8/15 WT/HT and 7/15 MT). Thus, we concluded that the specification of cranial NCCs was normal in *hmgcs1* mutant embryos. We next asked whether *hmgcs1* expression is necessary for migration of NCCs into the developing pharyngeal arches. To test this hypothesis, we measured the expression of RFP in *Tg*(*sox10*:TagRFP) [16,18] in mutant *hmgcs1* embryos or their siblings at 1 dpf between Prim-5 and Prim-15 stages. We consistently observed the formation of the pharyngeal arches in both siblings and homozygous mutants (Fig 2C & 2D, n = 20 15/20 WT/HT and 5/20 MT). Taken together these data suggest that specification and migration occur normally in the absence of complete *hmgcs1* function.

### *hmgcs1* modulates the differentiation of cranial neural crest cells

**Fig 3:**
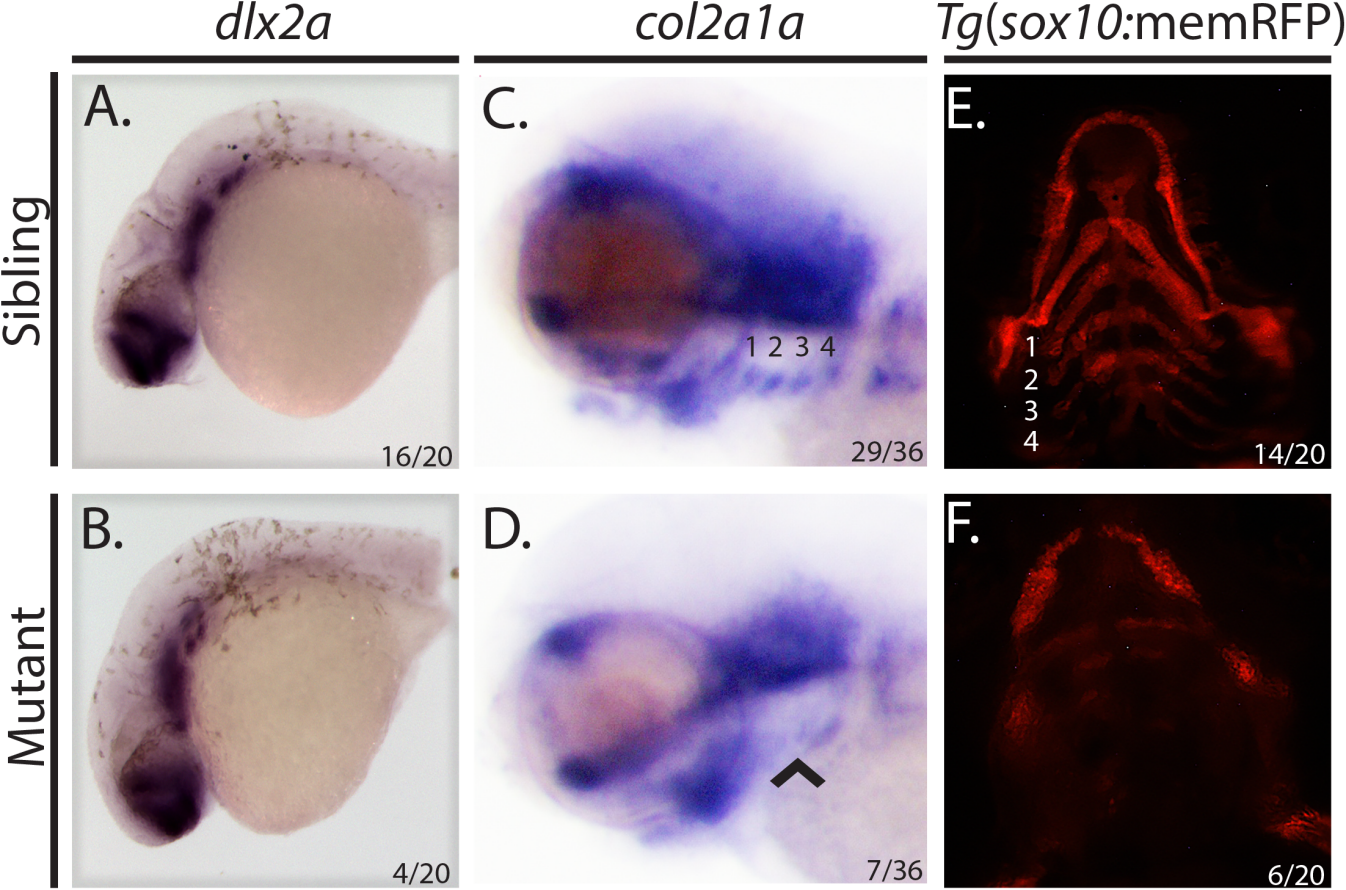
*hmgcs1* modulates cranial neural crest cell differentiation. (A&B) Whole mount *in situ* hybridization (ISH) was performed at 1 day post fertilization (dpf) between Prim-5 and Prim- 15 on wildtype/heterozyous *hmgcs1* siblings (Sibling) or *hmgcs1* homozygous mutant larvae (Mutant) using a riboprobe specific to *dlx2a*. n = 20 including 16/20 wildtype and heterozygous siblings and 4/20 homozygous mutants. Panel A depicts a wildtype sibling and no significant difference between wildtype embryos was detected. (C&D) ISH was performed at 3 dpf (protruding mouth stage) on *hmgcs1* siblings (wildtype and heterozygous individuals) or *hmgcs1* homozygous mutant larvae with a riboprobe specifically detecting *col2a1a* expression. Numbers denote the pharyngeal arch patterns present a 3dpf. Arrowhead denotes a loss of defined expression of *col2a1a* in *hmgcs1* mutant larvae. n = 36 including 29 wildtype and heterozygous individuals with 7 homozygous mutants. Panel C depicts a wildtype sibling with no significant differences observed between wildtype and heterozygous carriers. (E&F) *Tg*(*sox10*:memRFP) *hmgcs1* siblings or homozygous *hmgcs1* mutant larvae were imaged for RFP expression at 3dpf at the protruding mouth stage. Numbers indicate structures that will give rise to the putative ceratobranchial cartilages. n = 20 including 14/20 wildtype and heterozygous siblings and 6/20 homozygous mutants. Panel E depicts a wildtype sibling with no significant differences observed in heterozygous carriers.

Previous studies suggest that the cholesterol synthesis pathway modulates post-migratory NCC differentiation [5]. We therefore hypothesized that mutation of *hmgcs1* might interfere with cranial NCC differentiation. To test this, we performed *in situ* hybridization to detect the expression of *dlx2a* and *col2a1a* in wildtype siblings and *hmgcs1* mutant animals. Dlx2a is a transcription factor needed to produce and maintain cranial NCCs [25] and *col2a1a* encodes a component of type II collagen [26], both of which are markers of cranial NCC differentiation. *In situ* hybridization revealed normal staining of *dlx2a* in siblings and *hmgcs1* homozygous mutant animals in the pharyngeal arches and the developing forebrain at 1 dpf between Prim-5 and Prim-15. These data suggest that early cranial neural crest migration and development are normal in *hmgcs1* mutants (Fig 3A & 3B, n = 20, 16/20 WT/HT, 4/20 MT).

We next compared the expression of *col2a1a* in mutants and their wildtype siblings at 3dpf, the protruding mouth stage. At 3 dpf, *hmgcs1* mutants demonstrate with clear morphological deficits including cardiac edema, a smaller jaw, and an opaque brain [9]. Mutation of *hmgcs1* disrupted the expression of *col2a1a* in a spatially restricted manner causing an absence of defined *col2a1a* expression in the pharyngeal endoderm (Fig 3C & 3D, n = 36, 29/36 WT/HT, 7/36 MT). However, expression of *col2a1a* was still present in the oral ectoderm and ventral brain, although the oral region in mutant embryos was slightly truncated (Fig 3C & 3D). Finally, we measured the expression of RFP in the *Tg*(*sox10*:memRFP) reporter animal at 3 dpf at the protruding mouth stage to determine whether Sox10 expression was perturbed in regions lacking expression of *col2a1a*. Consistent with *col2a1a* expression patterns, RFP was expressed in the developing facial structures of siblings including regions that give rise to the Meckel’s cartilage, ceratohyal, and the ceratobranchial cartilages. However, *hmgcs1* mutants did not express the appropriate level of RFP in the regions which give rise to the ceratobranchial cartilages, the ceratohyal, and the basihyal (Fig 3E & 3F, n = 20, 14/20 WT/HT, 6/20 MT), indicating that late stage cranial NCCs fail to fully differentiate.

### Hmgcs1 modulates facial development by cholesterol dependent and independent mechanisms

**Fig 4:**
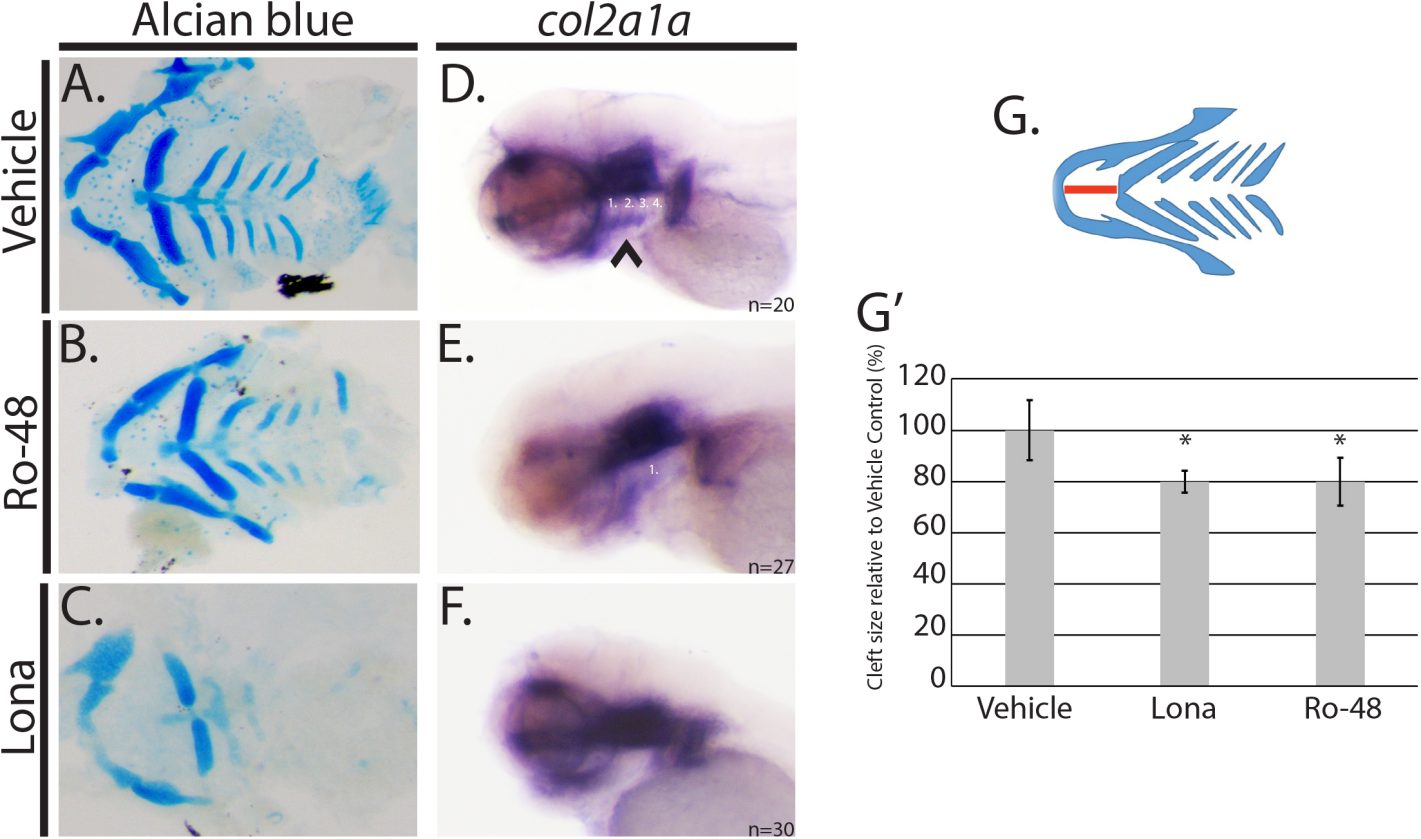
*hmgcs1* regulates facial development by a cholesterol independent mechanism. (A-C) Wildtype AB embryos were treated with 0.01% DMSO (vehicle), 8uM lonafarnib (Lona) or 3 uM Ro 48 8071 (Ro-48) beginning at 5 hours post fertilization and for a period of 4 days post fertilization (dpf). Alcian blue was used to analyze the cartilage structures of the developing zebrafish head and neck. (A-C) demonstrate the dissected viscerocranium of 4 day old larvae. (DMSO n = 30, Ro-48 n = 14, and Lona n = 8) (D-F) Whole mount *in situ* hybridization (ISH) was performed at 3 days post fertilization (protruding mouth stage) with embryos treated with 0.01% DMSO (vehicle), 8uM lonafarnib (Lona) or 2.5 uM Ro 48 8071 (Ro-48) (DMSO n = 20, Ro 48 n = 27, and Lona n = 30) (G) Schematic representation of viscerocranium. The extension of the Meckel’s cartilage (mandible) was measured as a detection for the presence of a facial cleft phenotype. Distance is indicated by the red line. (G’) Average was normalized and represented relative to the vehicle control group. Asterisk denotes statistical significance of p<0.001. Statistical analysis was performed using a T-test.

Given the presence of facial abnormalities in genetic disorders of cholesterol metabolism, we sought to determine if the facial deficits in *hmgcs1* mutants were cholesterol dependent. We first treated embryos with 2.5uM atorvastatin, which inhibits the rate limiting step of cholesterol synthesis pathway, abolishing both cholesterol and isoprenoids, effectively mimicking the biochemical phenotypes associated with mutation of *hmgcs1* and then analyzed the developing facial features at 4dpf with alcian blue. Animals treated with vehicle control (DMSO) did not exhibit any facial dysmorphia (Fig 4A), however treatment with atorvastatin resulted in the loss of ceratobranchial cartilages and the basihyal, a finding consistent with previously published work [5] (Table 1, n = 30, p-value<0.0001). Next, we inhibited cholesterol synthesis by treating embryos with varied concentrations of Ro 48 8071, which specifically inhibits cholesterol synthesis and not isoprenoid synthesis, in a dose dependent manner [9,27,28]. Embryos treated with 2uM or less Ro 48 8071 did not exhibit with craniofacial abnormalities (Table 1). However, at 2.5uM or higher, a significant percentage of embryos exhibited cranial facial abnormalities (Table 1). Inhibition of cholesterol with Ro 48 8071 also resulted in a truncation of the Meckel’s cartilage, stunted ceratobranchial cartilages or missing posterior ceratobranchial cartilage structures (Fig 4A & 4B, p<0.0001). The degree to which the Meckel’s cartilage is truncated has not been previously quantified. Therefore, we measured the extension of the Meckel’s cartilage from the base of the ceratohyal (Fig 4G). Embryos treated with a minimum of 2.5uM Ro 48 8071 (n = 14) had a mandible that was truncated by approximately 20% when compared to vehicle control (Fig 4G & 4G’). Importantly, concentrations that exceeded 3uM Ro 48 8071 resulted in embryonic lethality (Table 1). Defects observed in all Ro 48 8071 sub-groups were mild when compared with the defects present in *hmgcs1* mutant larvae (Fig 1).

**Table 1:** Inhibition of cholesterol and isoprenoids results in dose dependent craniofacial abnormalities.

Treatment	Total Embryos	Percent Survival (%)	Craniofacial abnormalities	p-value
DMSO	90	100	1	
Atorvastatin-2.5uM	30	100	23	<0.0001
Lonafarnib-8uM	40	95	35	<0.0001
Ro 48 8071–1.0uM	18	92	1	0.3069
Ro 48 8071–2.0uM	42	100	5	0.0125
Ro 48 8071–3.0uM	15	100	14	<0.0001
Ro 48 8071–4.0uM	25	0	N/A	N/A

Abnormal Phenotype includes truncated Meckel’s cartilage, presence or absence of an inverted ceratohyal, and the loss of the posterior ceratobranchial cartilages. Statistical analysis was performed with a Fisher’s Exact T-test.

The moderate nature of the phenotype associated with inhibition of cholesterol only, prompted us to ask whether isoprenoids have a function during craniofacial development. To address this hypothesis, we treated embryos with 8uM lonafarnib, which specifically inhibits the synthesis of farnesylated isoprenoids. Notably, concentrations exceeding 8uM resulted in embryonic lethality by 5 dpf. Treatment with 8uM lonafarnib (n = 8) resulted in a truncated Meckel’s cartilage, a linear ceratohyal, a missing basihyal, and diminished cartilage staining in the ceratobranchial cartilages (Fig 4A & 4C, p<0.0001). Inhibition of isoprenoid synthesis resulted in a comparable phenotype as was observed in *hmgcs1* mutant larvae and larvae treated with atorvastatin (Figs 1 & 4). Moreover, we quantified the degree of truncation of the Meckel’s cartilage structure in lonafarnib treated animals and found that treatment resulted in a near 20% truncation, similar to what was observed when embryos were treated with Ro 48 8071 (Fig 4G & 4G’ p<0.001). Additionally the severity of the phenotype was dose dependent, demonstrating more severe phenotypes at the highest concentrations (data not shown). Notably, the severity of the Meckel’s cartilage truncation was not associated with length of treatment, because 24 hours of treatment at the indicated concentrations was sufficient to truncate the structure by approximately 20%, an equivalent truncation compared with 4 days of continuous treatment (Figs 4 and S2). These data suggest that the cholesterol synthesis pathway regulates facial development by both cholesterol dependent and independent mechanisms.

### Cholesterol and isoprenoids regulate the differentiation of cranial NCCs

Our analysis has demonstrated that mutations in *hmgcs1* interfere with proper facial development by inhibiting the differentiation of cranial NCCs. Moreover, we have shown that facial development occurs via cholesterol dependent and cholesterol independent mechanisms. However, whether both branches of the pathway regulate the same step of development, cranial NCC differentiation, is not clear. To understand the function of cholesterol and isoprenoids during cranial NCC differentiation, we measured the expression of *col2a1a* in animals treated with 8uM lonafarnib, 2.5uM Ro 48 8071, or vehicle control at 3dpf, the protruding mouth stage. Embryos treated with vehicle control (DMSO) (n = 20), produced all structures of the viscerocranium appropriately (Fig 4A), expressed *col2a1a* in the ventral brain, oral ectoderm, and pharyngeal endoderm (Fig 4D). In contrast treatment with 8uM lonafarnib (n = 30) resulted in the loss of *col2a1a* in the pharyngeal endoderm, although expression remained consistent in the brain and oral ectoderm (Fig 4F). The loss of *col2a1a* in lonafarnib treated embryos was marked, however the loss of expression was more moderate in the Ro 48 8071 treatment group (n = 27), where expression was present in the in the pharyngeal endoderm, albeit with less definition (Fig 4E). These data are consistent with the alcian blue results observed in embryos undergoing pharmacological inhibition (Fig 4A–4C) and suggest that cholesterol and isoprenoids have overlapping functions during cranial NCC differentiation.

### *hmgcs1* mutants exhibit temporal restricted changes in Shh activity

**Fig 5:**
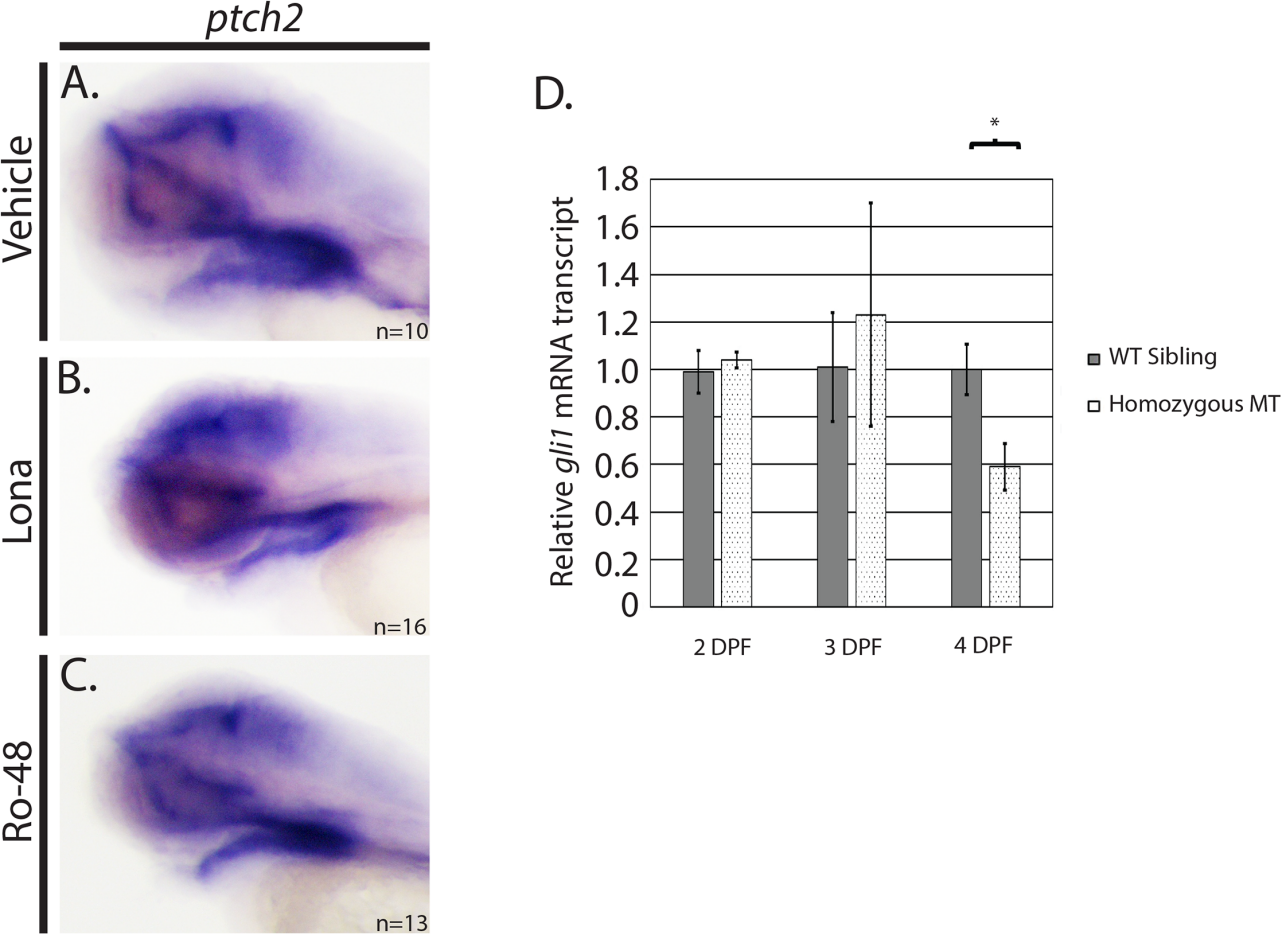
Inhibition of isoprenoids and cholesterol has not effect on sonic hedgehog (Shh) activity. (A-C) Whole mount *in situ* hybridization (ISH) was performed at 3 days post fertilization (protruding mouth stage) (dpf) with embryos treated with 0.01% DMSO (vehicle), 8uM lonafarnib (Lona) or 2.5 uM Ro 48 8071 (Ro-48). (DMSO n = 10, Ro-48 n = 13, and Lona n = 16). (D) Sybr green based quantitative real time PCR was performed on *hmgcs1* mutant embryos (Homozygous MT) or their wildtype siblings (WT Sibling) at the indicated days post fertilization (DPF) to detect mRNA expression of *gli1*. Error bars represent standard deviation of technical replicates.

Shh signaling is an important component of proper craniofacial development [13,29]. Furthermore, Shh signaling is mediated by post translational cholesterol modifications [30] and mutations in the proteins that facilitate these modifications have been associated with craniofacial abnormalities [31–33]. Given these results, we hypothesized that the cholesterol mediated craniofacial defects observed in *hmgcs1* mutants are Shh dependent. To test this hypothesis, we measured the expression of *ptch2*, a Shh receptor whose expression is activated upon Shh activity [34], in embryos treated with 8uM lonafarnib, 2.5uM at Ro 48 8071, or vehicle control at 3 dpf, the protruding mouth stage. *ptch2* RNA increases with active Shh signaling, therefore the expression of *ptch2* can be used as a marker of Shh activity [35]. *ptch2* expression was localized specifically to the facial region; present in the pharyngeal endoderm, oral ectoderm, and the brain of vehicle control embryos (Fig 5A, n = 10). Importantly, the localization of *ptch2* expression did not change significantly in embryos treated with either lonafarnib (Fig 5B, n = 16) or Ro 48 8071 (Fig 5C, n = 13) at the protruding mouth stage. These data are supported by similar experiments *hmgcs1* mutant larvae, as we did not observe a decrease in *ptch2* expression in mutant larvae at the protruding mouth stage by either *in situ* hybridization or quantitative real time PCR at 4 dpf (QPCR) (S3 Fig).

Decreased levels of *gli1* mRNA, the downstream effector of Shh, have been reported in a zebrafish mutant of *hmgcrb* at 52 hpf [5]. Increased *gli1* expression is a hallmark of active Shh signaling and *ptch* is a downstream target gene of the *gli1* transcription factor [36]. Thus, we performed QPCR at 2, 3, and 4 dpf to measure the level of *gli1* in *hmgcs1* mutants and their wildtype siblings. We did not detect a statistically significant difference in the expression of *gli1* in mutants or their wildtype siblings at 2 (long-pec) or 3 dpf (protruding mouth stage) (Fig 5D). However, we did detect an approximate 40% decrease in *gli1* expression at 4 dpf, a time point when maternal cholesterol level declines [9] (Fig 5D). Importantly, the decrease in *gli1* expression we detected occurred after the onset of craniofacial defects, as we observed defects in *col2a1a* expression at 3 dpf (protruding mouth stage) in *hmgcs1* mutants and embryos treated with Ro 48 8071 (Figs 3 and 4). These data strongly suggest that deficiencies in cholesterol synthesis cause craniofacial abnormalities prior to the onset of deficiencies in Shh signaling. Moreover, maternal cholesterol is sufficient to sustain Shh activity for up to 3 dpf in *hmgcs1* mutants.

## Discussion

NCCs develop into the cartilaginous structures of the neck and face. In order to accomplish this task, cells undergo a precisely controlled series of steps including specification, migration, proliferation, and differentiation [2]. Both positive and negative regulators of this process have been identified [37], but many mechanisms underlying this process have not yet been completely described. Cholesterol homeostasis has been implicated in the regulation of NCC development and vertebrate craniofacial development. Here we expand upon previously published work, by identifying the independent functions of cholesterol and isoprenoids during craniofacial development. Further, we confirm *in vivo*, that cholesterol plays a diverse and temporally regulated function during craniofacial development.

Mutation or inhibition of the cholesterol synthesis pathway in zebrafish causes multiple phenotypes including defects in central nervous system development, abnormal oligodendrocyte migration, cardiac edema, pigment defects, and craniofacial abnormalities [9]. Here we focus specifically on cranial NCCs, yet the presence of both cardiac and pigment deficits in zebrafish harboring mutations in this pathway provides strong evidence for a more global role of this pathway in NCC development. The function of the cholesterol synthesis pathway in NCC development must be subject to precise control because we found limited evidence that early neural crest specification or migration were defective in animals with mutation of *hmgcs1*. These data are strongly supported by a recent report characterizing the function of *hmgcrb*, the zebrafish ortholog of *HMGCR* in post migratory crest differentiation [5]. Despite the normality of early NCC function in *hmgcs1* mutant animals, we cannot completely rule out the possibility that the cholesterol synthesis pathway modulates early NCC development because there is significant maternal contribution of cholesterol in zebrafish until 4dpf [9], as observed when assaying for Shh activity in *hmgcs1* mutants. Moreover, *hmgcs1* mutants have documented pigment disorders and the mechanisms underlying such deficits are yet to be explored. Furthermore, the presence of several neural crest related human disorders such as Treacher Collins syndrome, Hirschsprung disease, and 22q11.2 deletion syndromes [38–41] suggests that there are inherent pathways that modulate the differentiation of numerous NCC lineages, perhaps in a time dependent manner.

Mutation of 7-dehydrocholesterol delta 7-reductase, the last enzyme in the cholesterol synthesis pathway causes SLOS [42]. Patients diagnosed with SLOS have reduced serum cholesterol which results in multiple congenital defects including intellectual disability, facial dysmorphism, and organ and limb anomalies [43]. SLOS is the result of a mutations in a gene, whose function is limited to cholesterol synthesis and to date has not been associated with defects in isoprenoid synthesis [44]. Thus, we hypothesized that the facial deficits present in animals harboring mutations in *hmgcs1* were a consequence of defects in cholesterol synthesis and independent of isoprenoid synthesis. We tested this hypothesis using pharmacological inhibition, which demonstrated that reduced cholesterol synthesis induced mild to moderate craniofacial deficits in a dose responsive manner. These data demonstrate that craniofacial development is in part mediated by cholesterol dependent mechanisms, however the severity of the craniofacial defects associated with cholesterol inhibition were moderate when compared to embryos with mutations in *hmgcs1*. These data raise the possibility that there are cholesterol independent mechanisms underlying the craniofacial deficits observed in *hmgcs1* mutant animals. We tested this hypothesis by specifically inhibiting isoprenoid synthesis, which demonstrated a novel and previously underappreciated role for isoprenoids during craniofacial development. We suspect that the moderate nature of the facial dysmorphia present in Ro 48 8071 treated embryos is a consequence of either maternal contributions of cholesterol [9].

Isoprenoids are essential for protein prenylation [45] and protein prenylation is a major component of cell signaling, specifically the activity of cdc42, Rho kinase, and Ras [46]. Aberrant signaling could affect a myriad of downstream pathways including the MAP kinase ERK signaling pathway, Wnt signaling, and PI3 kinase signaling. Some of which have recently been implicated in cholesterol regulation of oligodendrocytes [47]. Importantly, both Wnt and ERK signaling are associated with craniofacial development [48,49]. Furthermore, germline mutations in *RAS*, which result in RASopathies, are associated with craniofacial abnormalities [50,51]. Importantly, our data suggests that defects in isoprenoid synthesis have the potential to impact development on a global scale, including craniofacial development.

Our pharmacological studies confirm that craniofacial development is indeed regulated, at least in part, by cholesterol synthesis and these effects are dose dependent (Table 1). One possible mechanism by which cholesterol could exert such effects is by modulating Shh signaling. During lipid modification of hedgehog (Hh) ligands, cholesterol is linked to Hh by dispatched 1 (Disp1) [14]. Mutations in either the murine or telost *Disp1* genes result in craniofacial abnormalities [31,33]. Moreover, human mutations in the Shh pathway cause overlapping phenotypes when compared with mutations that interfere with the cholesterol synthesis pathway [29,52]. *In vitro* experiments also suggest that deficiencies in the cholesterol synthesis pathway reduce Shh signaling by interfering with smoothened activation, but very few studies have linked these two pathways *in vivo* [11]. Therefore, we sought to confirm the interaction between the cholesterol synthesis pathway and Shh signaling in zebrafish with mutations in *hmgcs1*. We analyzed the expression of *ptch2*, the receptor of Shh, whose expression provides an indication of active Shh signaling, but did not observe significant defects in *ptch2* expression at 3dpf (protruding mouth stage) or 4 dpf suggesting that Shh activity is relatively normal at these developmental time points in *hmgcs1* mutants. Moreover, we measured the expression of *gli1* mRNA at 2 (long-pec), 3 (protruding mouth stage), and 4 dpf. *gli1* expression was normal at 2 and 3 dpf, but was decreased at 4 dpf, a time point when the vast majority of the maternal cholesterol has been depleted [9]. This temporal contribution of maternal cholesterol is highly significant because maternal cholesterol appears to be sufficient to promote effective Shh signaling at 2 and 3 dpf and is able to prevent significant morphological abnormalities from occurring very early in development. However, despite maternal contribution, our analysis revealed defects in chondrocyte differentiation at 3 dpf in *hmgcs1* mutants and in Ro 48 8071 treated embryos. Both *gli1* and *ptch2* expression was normal at 3dpf, according to QPCR and *in situ* hybridization, suggesting that the cholesterol related deficiencies we observed in facial development occur prior to the onset of defects in Shh signaling. Thus, cholesterol most likely regulates facial development through a combination of Shh dependent and independent mechanisms. One possible Shh independent mechanism includes the accumulation of sterols, which has been documented in *Insig* knockout mice [10]. Future experiments that decipher the pathways affected by cholesterol deficiency are needed to fully understand the function of cholesterol in facial development.

Our data analyzing Shh activity are strongly supported by previously published work except that mutations in *hmgcrb* are associated with minor *gli1* abnormalities at 52 hpf [5]. There are several putative explanations for the differences observed in our data sets. For example, the mutations in both *hmgcs1* or *hmgcrb*, are single missense mutations and could encode proteins with some degree of residual activity [5,9]. Moreover, each protein may be associated with different phenotypic penetrance or perhaps the maternal contribution from each zebrafish line are slightly variable, all of which could account for the differences observed in *gli1* expression. Future studies that quantitate the degree of Shh activity in different disorders of post-squalene cholesterol biosynthesis are needed to fully understand the interplay between Shh and the cholesterol synthesis pathway.

Here we formally demonstrate a function for the cholesterol synthesis pathway in facial development, specifically we demonstrate that facial deficits are the consequence of not only deficiencies in cholesterol, but also isoprenoid synthesis. Further, we find Shh signaling is intact in *hmgcs1* mutants at 2 and 3 dpf, although craniofacial abnormalities are evident by 3 dpf. These data provide some degree of evidence that there are Shh independent mechanisms downstream of cholesterol, which are critically important for craniofacial development between the long-pec and protruding mouth stage of zebrafish development. Future studies deciphering the downstream pathways modulated by cholesterol and isoprenoids will be necessary to gain a more comprehensive understanding of neural crest associated human diseases.

## Supporting information

S1 Fig Vu57 Siblings do not demonstrate with orofacial clefts. Wildtype and heterozygous Vu57 siblings were subjected to alcian blue staining at 4 days post fertilization. The distance between Meckel’s cartilage and the ceratohyal were measured to detect potential mandible cleft phenotype. Error bars indicate standard deviation. No significant difference was detected between wildtype and heterozygous individuals. Samples were compared using a standard T-test.
(TIF)

S2 Fig Orofacial development is temporally regulated by the products of the cholesterol synthesis pathway. Wildtype embryos were treated with Vehicle control (DMSO), 8uM lonafarnib, or 2.5uM Ro-48-8071 to inhibit farnesylated isoprenoids and cholesterol, respectively. Drugs were administered at 24 hours post fertilization and removed after 24 hours of treatment. Orofacial clefts were apparent based upon the distance between Meckel’s cartilage and the developing ceratohyal at 4 days post fertilization. Distance was measured in embryos stained with alcian blue. Each treatment resulted in a statistically significant orofacial cleft (p-value<0.001) using a standard T-test. Error bars indicate standard deviation.
(TIF)

S3 Fig Mutations in *hmgcs1* do not affect sonic hedgehog signaling. A-B. Whole mount *in situ* hybridization was performed on wildtype and mutant siblings at 3 days post fertilization/protruding mouth stage to detect the expression of *ptch2*. n = 47 including 17 wildtype, 7 homozygous mutants, and 23 heterozygous mutants. C. Real time PCR was performed on a pool (n = 20) of siblings or *hmgcs1* mutant embryos at 4 days post fertilization to detect *ptch2* expression (ptc2). Error bars demonstrate the standard error of the mean across two biological replicates each with 20 embryos per pool.
(JPG)
