# Supplementary figures and images for: Functional analysis of the zebrafish ortholog of HMGCS1 reveals independent functions for cholesterol and isoprenoids in craniofacial development
Source: PLoS One. 2017 Jul 7;12(7):e0180856. doi: 10.1371/journal.pone.0180856 (PMC5501617; doi:10.1371/journal.pone.0180856)

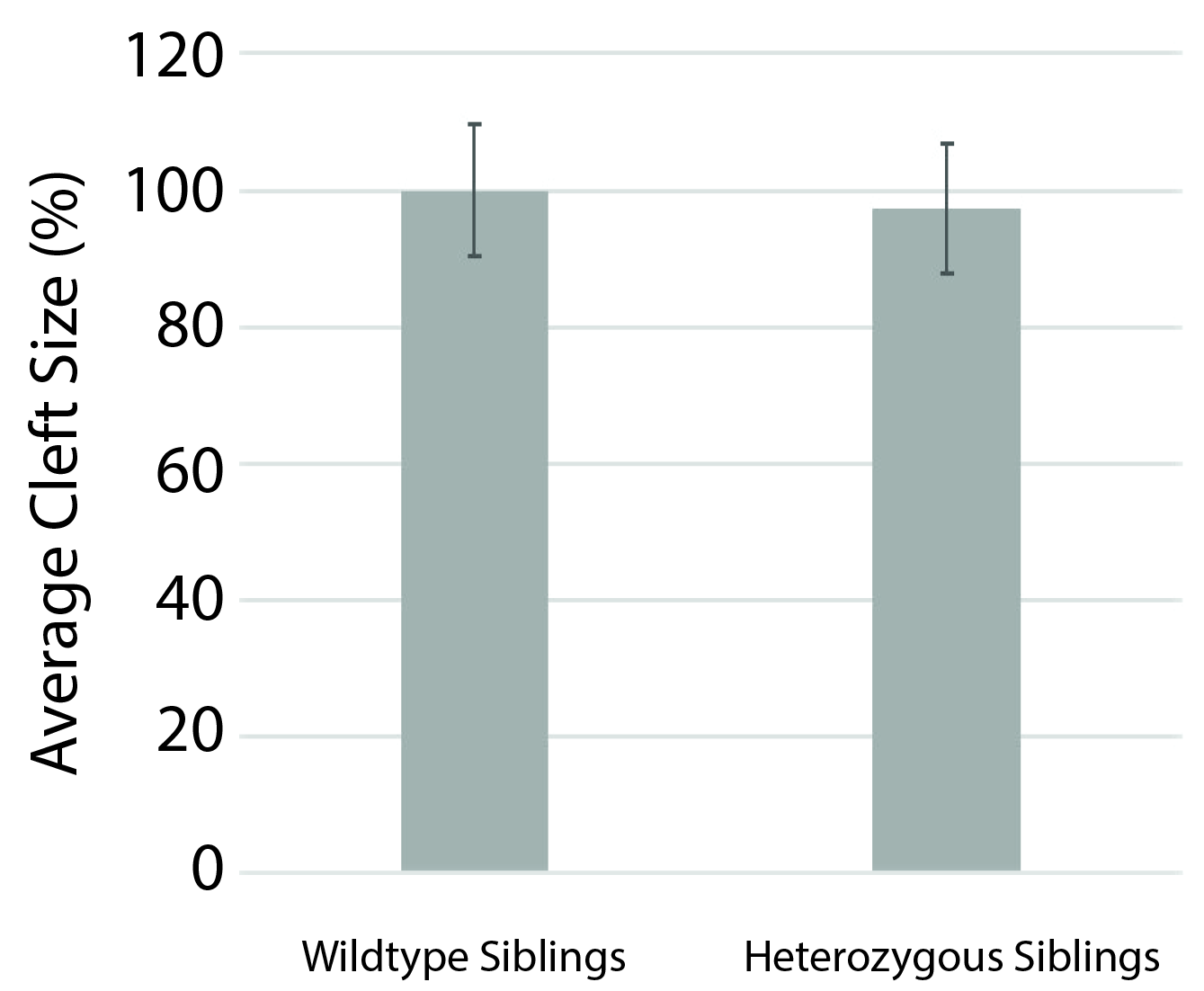

Supplement: S1 Fig — Wildtype and heterozygous Vu57 siblings were subjected to alcian blue staining at 4 days post fertilization. The distance between Meckel’s cartilage and the ceratohyal were measured to detect potential mandible cleft phenotype. Error bars indicate standard deviation. No significant difference was detected between wildtype and heterozygous individuals. Samples were compared using a standard T-test. (TIF) [file pone.0180856.s001.tif]

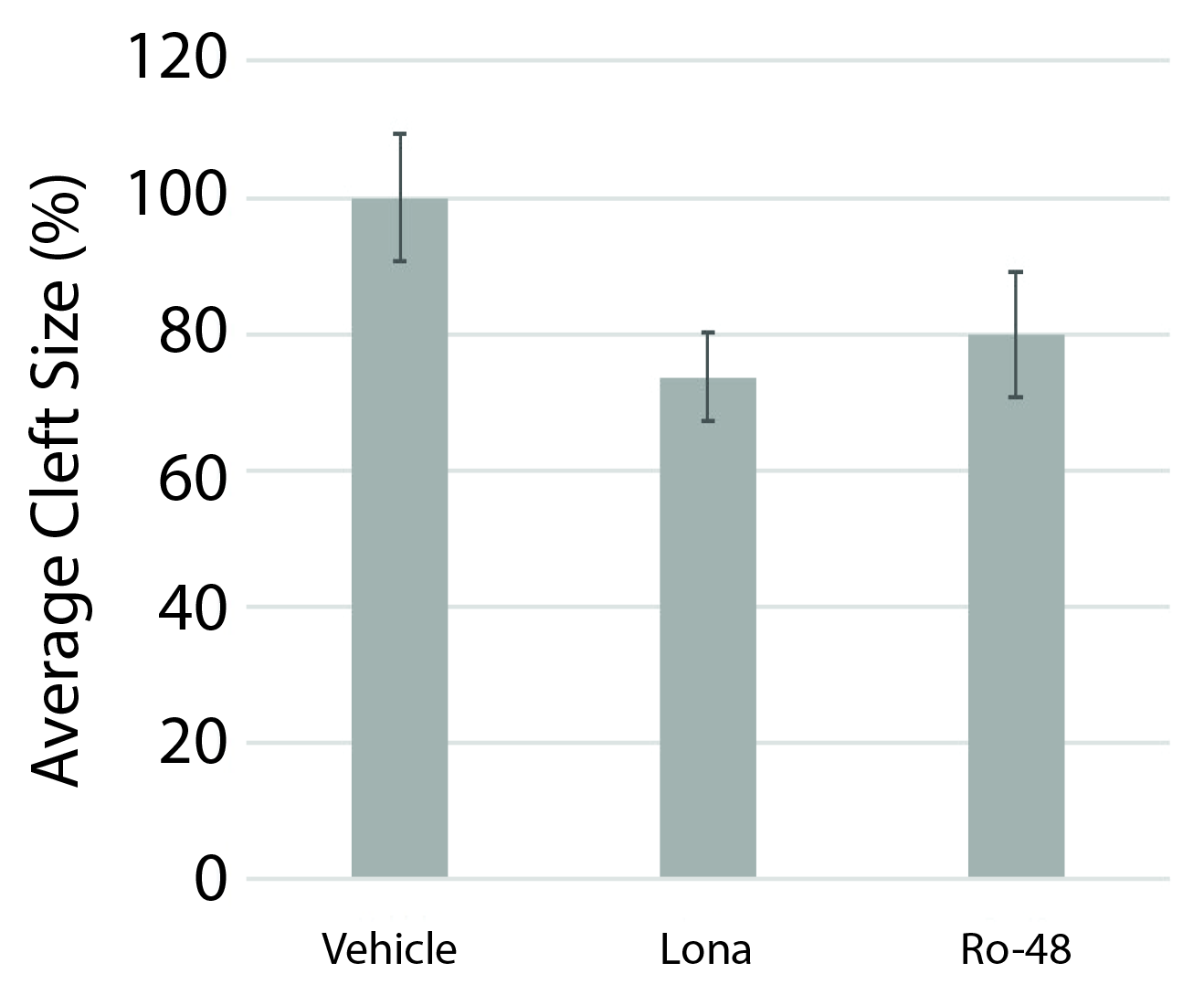

Supplement: S2 Fig — Wildtype embryos were treated with Vehicle control (DMSO), 8uM lonafarnib, or 2.5uM Ro-48-8071 to inhibit farnesylated isoprenoids and cholesterol, respectively. Drugs were administered at 24 hours post fertilization and removed after 24 hours of treatment. Orofacial clefts were apparent based upon the distance between Meckel’s cartilage and the developing ceratohyal at 4 days post fertilization. Distance was measured in embryos stained with alcian blue. Each treatment resulted in a statistically significant orofacial cleft (p-value<0.001) using a standard T-test. Error bars indicate standard deviation. (TIF) [file pone.0180856.s002.tif]

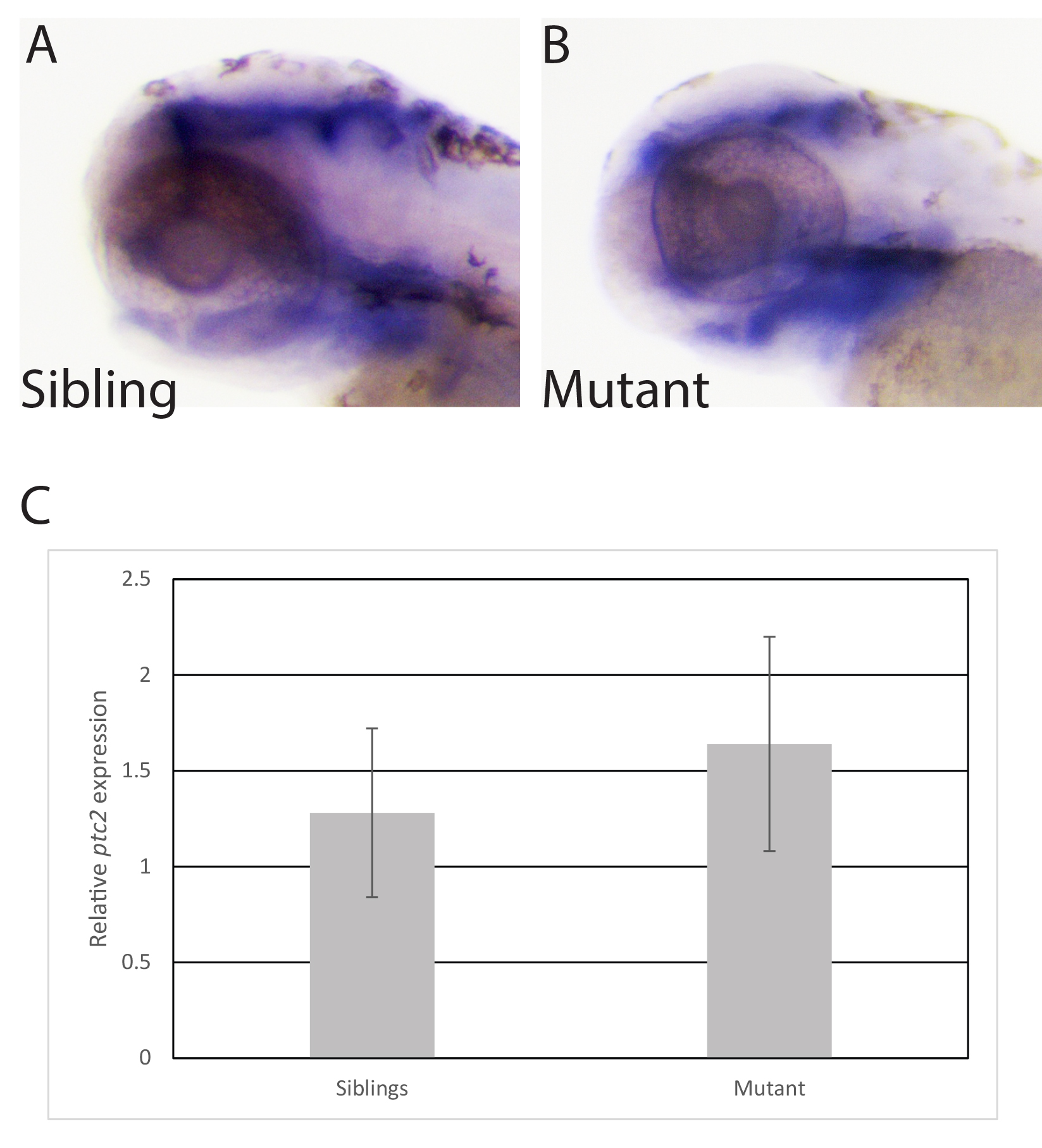

Supplement: S3 Fig — A-B. Whole mount in situ hybridization was performed on wildtype and mutant siblings at 3 days post fertilization/protruding mouth stage to detect the expression of ptch2. n = 47 including 17 wildtype, 7 homozygous mutants, and 23 heterozygous mutants. C. Real time PCR was performed on a pool (n = 20) of siblings or hmgcs1 mutant embryos at 4 days post fertilization to detect ptch2 expression (ptc2). Error bars demonstrate the standard error of the mean across two biological replicates each with 20 embryos per pool. (JPG) [file pone.0180856.s003.jpg]
